# CRISPR-guided base editor enables efficient and multiplex genome editing in bacterial cellulose-producing *Komagataeibacter* species

**DOI:** 10.1128/aem.02455-24

**Published:** 2025-01-31

**Authors:** Bo Xin, Jiaheng Liu, Jinyang Li, Zhaojun Peng, Xinyue Gan, Yuxi Zhang, Cheng Zhong

**Affiliations:** 1 State Key Laboratory of Food Nutrition & Safety, College of Biotechnology, Tianjin University of Science and Technology 577926 , Tianjin, China; 2 Key Laboratory of Industrial Fermentation Microbiology (Ministry of Education), Tianjin University of Science and Technology 562586 , Tianjin, China; Kyoto University, Kyoto, Japan

**Keywords:** CRISPR, base editing, genome editing, cytidine deaminase, bacterial cellulose, *Komagataeibacter*

## Abstract

Bacterial cellulose (BC) is an extracellular polysaccharide produced by bacteria that has wide applications in the food industry, tissue engineering, and battery manufacturing. Genome editing of BC-producing *Komagataeibacter* species is expected to optimize BC production and its properties. However, the available technology can target only one gene at a time and requires foreign DNA templates, which may present a regulatory hurdle for genetically modified organisms. In this study, we developed a clustered regularly interspaced short palindromic repeats (CRISPR)-guided base editing method for *Komagataeibacter* species using Cas9 nickase and cytidine deaminase. Without foreign DNA templates, C-to-T conversions were performed within an 8 bp editing window with 90% efficiency. Double- and triple-gene editing was achieved with 80%–90% efficiency. Fusing uracil-DNA glycosylase with the base editor enabled C-to-G editing. The base editor worked efficiently with various *Komagataeibacter* species. Finally, mannitol metabolic genes were investigated using base-editing-mediated gene inactivation. This study provides a powerful tool for multiplex genome editing of *Komagataeibacter* species.

**IMPORTANCE:**

*Komagataeibacter*, a bacterial genus belonging to the family *Acetobacteraceae*, has important applications in food and material biosynthesis. However, the genome editing of *Komagataeibacter* relies on traditional homologous recombination methods. Therefore, only one gene can be manipulated in each round using foreign DNA templates, which may present a regulatory hurdle for genetically modified organisms when microorganisms are used in the food industry. In this study, a powerful base editing technology was developed for *Komagataeibacter* species. C-to-T and C-to-G base conversions were efficiently implemented at up to three loci in the *Komagataeibacter* genome. This base editing system is expected to accelerate basic and applied research on *Komagataeibacter* species.

## INTRODUCTION

Bacterial cellulose (BC), composed of glucose molecules linearly linked with β-1,4-glycosidic bonds, is an extracellular polysaccharide synthesized by several kinds of bacteria (1–3). BC has excellent properties, such as high-degree polymerization, high mechanical strength, high hydrophilicity, superfine mesh structure, and good thermal stability and biocompatibility. Therefore, it is widely used in biodegradable packaging, food stabilizers, functional food ingredients, tissue engineering, wound therapy, cosmetics, battery manufacturing, sewage treatment, and papermaking (4–10).

Acetic acid bacteria of the *Komagataeibacter* genus are efficient BC producers capable of utilizing various renewable agricultural and marine feedstocks. The BC biosynthetic mechanism of *Komagataeibacter xylinus* has been revealed and studied (11). Glucose is first oxidized to gluconate by a membrane-bound glucose dehydrogenase. Gluconate is transported into the cells and enters the pentose phosphate pathway for catabolism and carbon rearrangement. Membrane-bound BC synthase complex catalyzes the final step of BC biosynthesis using UDP‐glucose as a substrate and secretes BC into the extracellular environment (12). To optimize substrate utilization, enhance BC production, and customize the BC structural property of *K. xylinus*, genetic manipulation is required (11). To this end, lambda Red and FLP/FRT-mediated homologous recombination systems have been used for gene deletion in *K. xylinus* (13). Nevertheless, providing plasmid-borne templates is a mandatory requirement and only one gene can be manipulated each time. Therefore, the development of a template-free multiplex genome editing method for *Komagataeibacter* is highly desirable.

Base editing combines a catalytically impaired Cas mutant and a nucleobase deaminase to introduce point mutations at target loci without introducing double-stranded DNA breaks (DSBs), adding exogenous DNA, or depending on homologous recombination (14). A typical cytosine base editor consists of a catalytically impaired Cas mutant and a cytidine deaminase to catalyze the deamination of C to U at the target site. The U:G heteroduplex is then converted into a T:A base pair following polymerase PolIII-mediated DNA replication, generating a C-to-T transition. In the case of U excision by uracil-DNA glycosylase (UNG), an apurinic/apyrimidinic (AP) site is formed and then repaired by the translesion DNA synthesis (TLS) pathway, producing C-to-A or C-to-G transversions in different hosts (15, 16). Although base editing was originally developed to cure genetic diseases (17, 18), it has been adapted for the genetic engineering of plants and microorganisms (19–23). Eliminating the requirement for foreign DNA templates simplifies the construction of editing plasmids and subsequent genome editing processes. Moreover, base editing may circumvent genetically modified organism regulations, which would be advantageous for industrial applications, especially in the food industry (19, 24).

In this study, we developed base editing tools for the efficient and multiplex genome editing of various *Komagataeibacter* species. Two and three targets were simultaneously edited with 80%–90% efficiency. Controllable C-to-T and C-to-G conversions were achieved using different base editor constructs. The developed base editing system serves as a powerful tool for gene inactivation by generating a premature stop codon inside the target gene and covers 93.5% (2,950 out of 3,155 genes) of *K. xylinus* CGMCC 2955 genes. As a demonstration, base editing was used to investigate mannitol metabolism, a major carbohydrate in seaweeds (25), providing useful information for strain engineering for BC production from marine feedstocks. This study enriches the genome editing toolbox for *Komagataeibacter* species and will accelerate the engineering of BC-hyperproducing strains.

## RESULTS AND DISCUSSION

### Programmable cytosine base editing in *K. xylinus*

**Fig 1:**
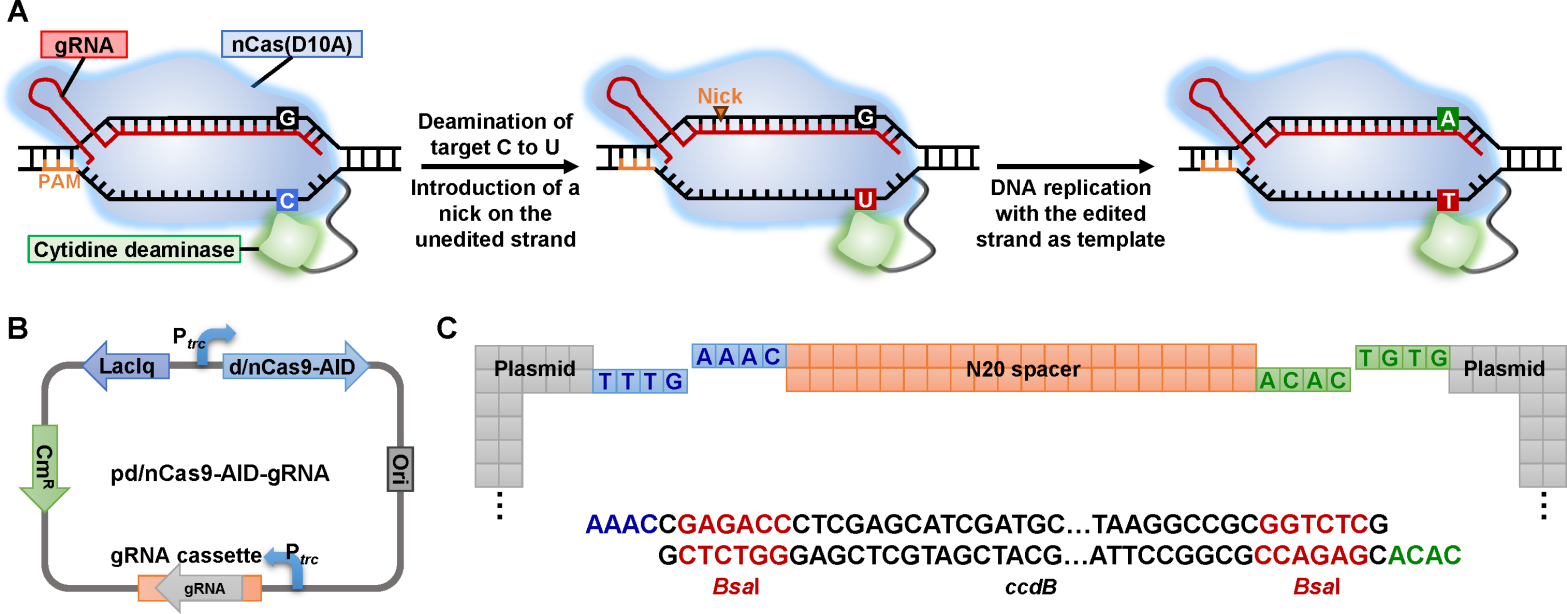
The base editing system developed for *Komagataeibacter*. (**A**) Schematic representation of the mechanism of C-to-T base editing by nCas9(D10A)-AID fusion guided by gRNA. Cytosine deamination by AID results in a U:G heteroduplex. nCas9(D10A) nicks the non-edited strand containing the G, favoring the resolution of the U:G heteroduplex to the desired T:A outcome. (**B**) Plasmid used for base editing in *Komagataeibacter*. (**C**) Schematic representation of the gRNA assembly strategy with a pair of 24 bp primers and Golden Gate assembly reaction.

To develop an effective base editing system in *Komagataeibacter*, we cloned two Target-activation-induced cytidine deaminase (AID) cytosine base editor constructs (18). The first one comprised a fusion of dCas9 (a nuclease-deficient mutant of *Streptococcus pyogenes* Cas9) and an AID ortholog (sea lamprey PmCDA1), and the second one comprised a fusion of nCas9(D10A) (a nickase capable of introducing a single-stranded break on the complementary strand) and the same AID (Fig. 1A). The d/nCas9-AID fusion genes were inserted into plasmid pSEVA331 under the control of isopropyl β-d-thiogalactoside (IPTG) inducible P*_trc_* promoter (26). Expression of the single guide RNA (gRNA) was controlled by a second P*_trc_* promoter (Fig. 1B). For convenient gRNA cassette construction, the 20 bp spacer region in the gRNA cassette was replaced by a *Bsa*I-*ccdB-Bsa*I cassette, facilitating easy gRNA cassette construction with a pair of 24 bp primers and the Golden Gate assembly reaction (Fig. 1C).

**Fig 2:**
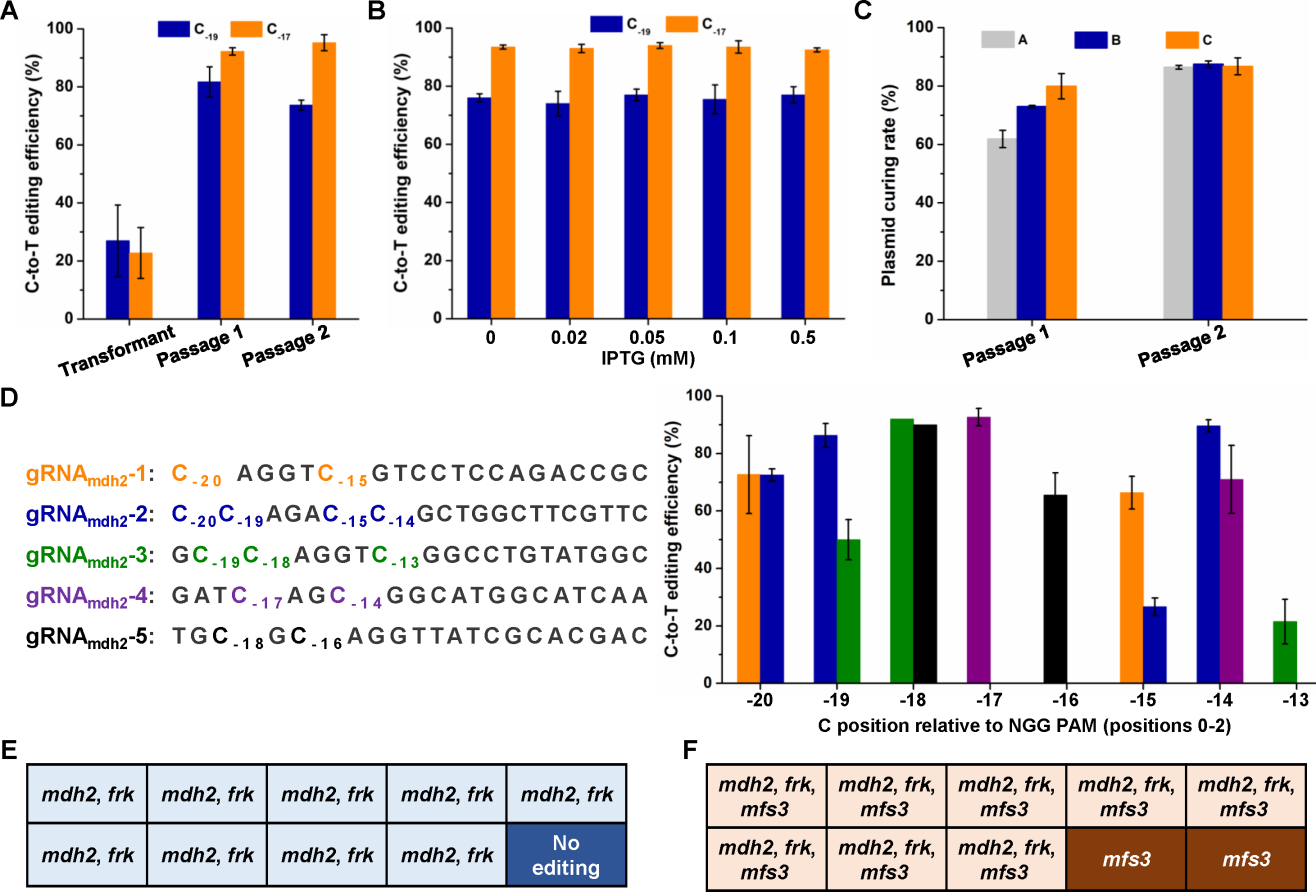
Characterization of the nCas9(D10A)-AID base editing tool in *K. xylinus*. (**A**) Base editing efficiency of different culture passages of transformants with nCas9(D10A)-AID-gRNA_frk_ plasmid. Error bars indicate standard deviations from three parallel experiments. Representative Sanger sequencing peaks for base editing in the transformant, passage 1, and passage 2 are shown in Fig. S1. (**B**) Effects of different IPTG concentrations on base editing efficiency. Error bars indicate standard deviations from three parallel experiments. Representative Sanger sequencing peaks for base editing with different IPTG usages are shown in Fig. S2. (**C**) Plasmid curing efficiency of edited cells with different cultivation passages. A, B, and C represent three independent colonies. Error bars indicate standard deviations from three parallel experiments. (**D**) Base editing targeting Cs at different positions in the spacer region. Error bars indicate standard deviations from three parallel experiments. (**E**) Double-gene editing by nCas9(D10A)-AID in *K. xylinus*. Ten colonies were randomly selected to amplify the target region of *mdh2* and *frk*. The PCR products were sequenced to evaluate the double-gene editing. *mdh2* and *frk* in the box represent the simultaneous editing of these two genes. Representative Sanger sequencing peaks for the double-gene editing colonies are shown in Fig. S4. (**F**) Triple-gene editing by nCas9(D10A)-AID in *K. xylinus*. Ten colonies were randomly selected to amplify the target region of *mdh2*, *frk,* and *mfs3*. The PCR products were sequenced to evaluate the triple-gene editing. *mdh2*, *frk*, and *mfs3* in the box represent the simultaneous editing of these three genes. For the rest two colonies, only *mfs3* was edited. Representative Sanger sequencing peaks for the triple-gene editing colonies are shown in Fig. S5.

To test the constructed base editing tools, a gRNA_frk_ was designed to target the chromosome of *K. xylinus* CGMCC 2955. Target-AID base editors have an approximately 5 bp editing window of protospacer positions −20 to −16 upstream of the NGG protospacer adjacent motif (PAM), counting the NGG PAM as positions 0–2 (27). Based on this analysis, there are two targetable Cs at positions −19 and −17 within the spacer region of gRNA_frk_ (Fig. 2A). The gRNA_frk_ was inserted into the dCas9-AID and nCas9(D10A)-AID expression plasmids. The resulting plasmids were then transfected into *K. xylinus* CGMCC 2955 and the transformants grown on agar plates with antibiotics were directly subjected to colony PCR to amplify the gRNA_frk_-targeting region. After Sanger sequencing the PCR products, the base editing efficiency was analyzed. Transformants of the dCas9-AID-gRNA_frk_ plasmid showed no detectable C-to-T conversions, whereas those of the nCas9(D10A)-AID-gRNA_frk_ plasmid showed editing efficiencies of 22.8% and 27.0% for C_−17_ and C_−19_, respectively (Fig. 2A; Fig. S1). Because IPTG was not added for induction, the observed base editing could be due to leaky expression of the P*_trc_* promoter. Transformants were then cultured on agar plates with both antibiotics and IPTG (0.1 mM) for inducing base editor expression (first passage). Colony PCR and Sanger sequencing were performed on the newly obtained colonies. dCas9-AID-gRNA_frk_ did not produce C-to-T conversions and thus was not investigated in the subsequent experiment. The C-to-T editing efficiencies of nCas9(D10A)-AID-gRNA_frk_ increased to 81.8% and 92.3% for C_−19_ and C_−17_, respectively. The colonies were used to inoculate new agar plates with antibiotics and IPTG (second passage). However, the second passage did not show a higher editing efficiency (Fig. 2A; Fig. S1).

Next, we tested the effects of IPTG on base editing. The transformants of the nCas9(D10A)-AID-gRNA_frk_ plasmid were used to inoculate agar plates with antibiotics and different concentrations of IPTG (0–0.5 mM). The colonies were used as templates for PCR amplification of the target region. The Sanger sequencing results showed that even without IPTG, the C-to-T editing efficiency reached 76.0% and 93.5% for C_−19_ and C_−17_, respectively (Fig. 2B; Fig. S2). Addition of IPTG did not increase the editing efficiency, suggesting that the leaky expression level of the base editor was sufficient to introduce target base conversions. This result was not surprising because the transformants of the nCas9(D10A)-AID-gRNA_frk_ plasmid on agar plates with only antibiotics already showed substantial base editing events with efficiencies over 20% (Fig. 2A).

### Easy curing of base editing plasmid

Immediate removal of the plasmid from the edited cells can reduce potential off-target editing caused by the clustered regularly interspaced short palindromic repeats (CRISPR) system. Plasmid curing is required for continuous strain engineering and industrial applications. Therefore, the three edited colonies were cultured in an antibiotic-free medium. When the optical density at 600 nm (OD_600_) reached approximately 1.0, the cells were spread on agar plates with or without antibiotics. The number of colonies was calculated to determine the frequency of plasmid loss. With only one culture passage, the plasmid curing frequency exceeded 60%. The first passage was then transferred to a fresh antibiotic-free medium for the second passage. Using the same assay, the plasmid-curing efficiency increased to over 90% (Fig. 2C). Finally, colonies were randomly selected to test their sensitivity to antibiotics to verify the removal of the plasmid. Besides, the target region for base editing was amplified. Sanger sequencing of the PCR product showed that the target region was fully edited and the colony isolated after plasmid curing was a pure culture (Fig. S3). This result suggests that the plasmid used for base editing is unstable without antibiotics, which is advantageous for easy plasmid curing.

### Examination of the target window for base editing

The two Cs at positions −17 and −19 upstream of the PAM sequence were both efficiently edited by the developed base editor. To test the editing window, we designed five gRNAs targeting a *mdh2* gene (gRNA_mdh2_-1–gRNA_mdh2_-5) that covered the canonical 5 bp editing window (−16 to −20 positions) and several Cs extending to the −13 position (Fig. 2D). Editing efficiencies for targeting Cs at different positions were determined by Sanger sequencing the target region after editing. The results suggest that all the Cs within the canonical 5 bp editing window (−16 to −20 positions) can be edited by the nCas9(D10A)-AID base editor with efficiencies of over 60%. Interestingly, Cs proximal to the canonical 5 bp editing window were also susceptible to base editing. Cs at positions −15 and −14 were edited with efficiencies of over 60%, and an editing efficiency of approximately 20% was observed for the C at position −13 (Fig. 2D). A wide target window is a highly desirable property that can increase the number of editable targets in a genome.

### Multiplex base editing for double and triple gene editing

Genome editing technology based on lambda Red and the FLP/FRT system can only edit one target gene each time in *K. xylinus*, which is mainly due to the requirement of donor DNA templates and homologous recombination (13). Conversely, neither DNA donor nor homologous recombination is required for base editing, suggesting the potential for multiplex genome editing (28, 29). Therefore, we performed double- and triple-gene editing in *K. xylinus* by simultaneously expressing gRNAs targeting two and three genes. After one passage, 10 colonies were randomly picked and sequenced to evaluate the multiplex genome editing efficiency. For double-gene editing, the target genes *mdh2* and *frk* were simultaneously edited in nine out of ten colonies, making the double-gene editing efficiency of 90%. The remaining single colony was not edited (Fig. 2E; Fig. S4). For triple-gene editing, the target genes *mdh2*, *frk*, and *mfs3* were simultaneously edited in eight out of ten colonies, resulting in a triple-gene editing efficiency of 80%. For the remaining two colonies, only *mfs3* was edited (Fig. 2F; Fig. S5). High-efficiency multiplex genome editing makes the developed base editing method a promising strategy for accelerating gene function analysis and strain engineering in *K. xylinus*.

### Off-target effects of the developed base editing tool

Although CRISPR-guided base editing has been suggested to have fewer off-target mutations than CRISPR/Cas9-based DSB-induced genome editing (17), previous base editing applications in microorganisms have resulted in a small number of off-target mutations in the chromosome (28, 30). To evaluate the off-target effect of the developed base editor in *K. xylinus*, the genomes of six base-edited *K. xylinus* strains were analyzed by next-generation sequencing (NGS). Besides the targeted base conversions introduced by the base editor, 21 single nucleotide variants were detected, all of which were C·G to T·A conversions (Table S1). One edited strain has no off-target mutations and another one has the most six off-target mutations. Because AID acts on single-stranded DNAs that may be available during DNA replication, these extra mutations are likely the result of random AID activity. However, we cannot exclude the possibility that these mutations occurred spontaneously during the experiment. For comparison, 22 single nucleotide polymorphisms (SNP) were detected in five base-edited *Corynebacterium glutamicum* strains, and 19 SNPs were detected in eight base-edited *Bacillus subtilis* strains (28, 30), which are similar to the results of the present study. For genetically engineered microorganisms used in the agricultural and food industries, low-frequency random mutations are not as critical as in base editing-mediated genetic therapy, which has strict requirements for avoiding off-target mutations. To reduce the adverse effect of nCas9(D10A)-AID overexpression, more stringently controlled inducible promoters can be used to limit the leaky expression of the base editor.

### Genome-wide analysis of base editing targets for gene inactivation

**Fig 3:**
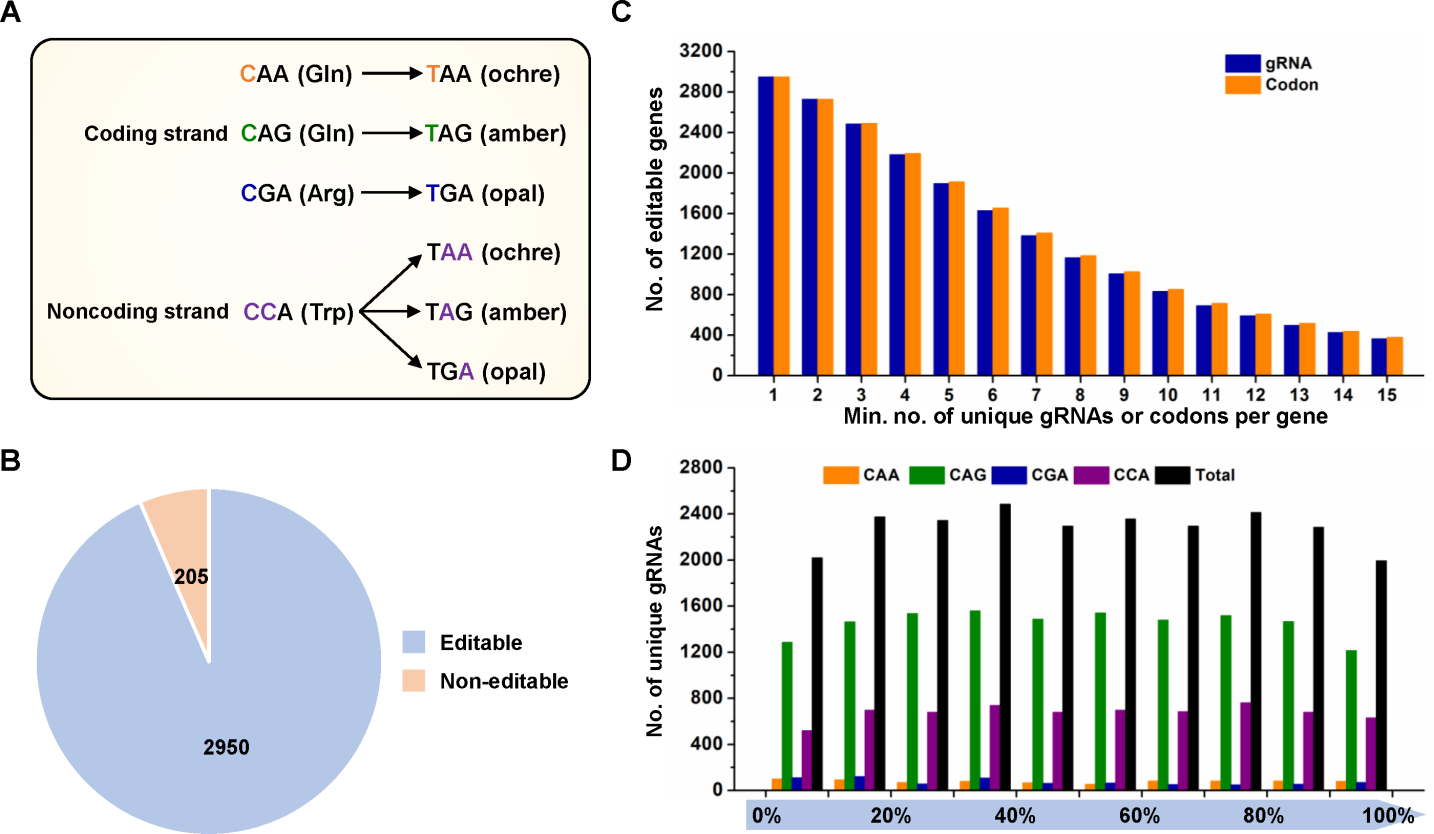
Genomic analysis of the base editing targets for gene inactivation in the *K. xylinus* genome. (**A**) Schematic representation of the C-to-T base editing-mediated introduction of stop codons. (**B**) Statistics of editable genes with at least one targetable codon for gene inactivation in the *K. xylinus* genome. (**C**) Number of editable genes with at least one available gRNA or editable codon for gene inactivation. (**D**) Relative positions of the editable codons for gene inactivation inside a gene.

C-to-T conversions introduced by nCas9(D10A)-AID into a target gene can be used to generate premature stop codons, which can lead to the inactivation of the target gene similar to CRISPR-mediated gene deletion. In the coding strand of a target gene, the CGA (Arg), CAG (Gln), and CAA (Gln) codons can be converted to TGA (opal), TAG (amber), and TAA (ochre) stop codons, respectively, using the C-to-T base editor. In addition, C-to-T conversions in either one or both Cs of CCA in the non-coding strand can create TGA, TAG, or TAA stop codons in the coding strand of the target gene (Fig. 3A). To determine the coverage of the base editor in the *K. xylinus* CGMCC 2955 genome, we performed a bioinformatics analysis using the gBIG tool (http://gbig.ibiodesign.net/) (27). In total, 22,870 unique gRNAs that can be used for gene inactivation were identified. Using these gRNAs, 2,950 of the total 3,155 genes (93.5%) can be deactivated by introducing premature stop codons (Fig. 3B; Table S2). Over 2,000 genes possessed at least four available gRNAs and editable codons, providing sufficient choices for gene inactivation (Fig. 3C). In addition, these codons were uniformly distributed within the coding region of the target genes (Fig. 3D).

### Expansion of the base editing type with glycosylase base editor

**Fig 4:**
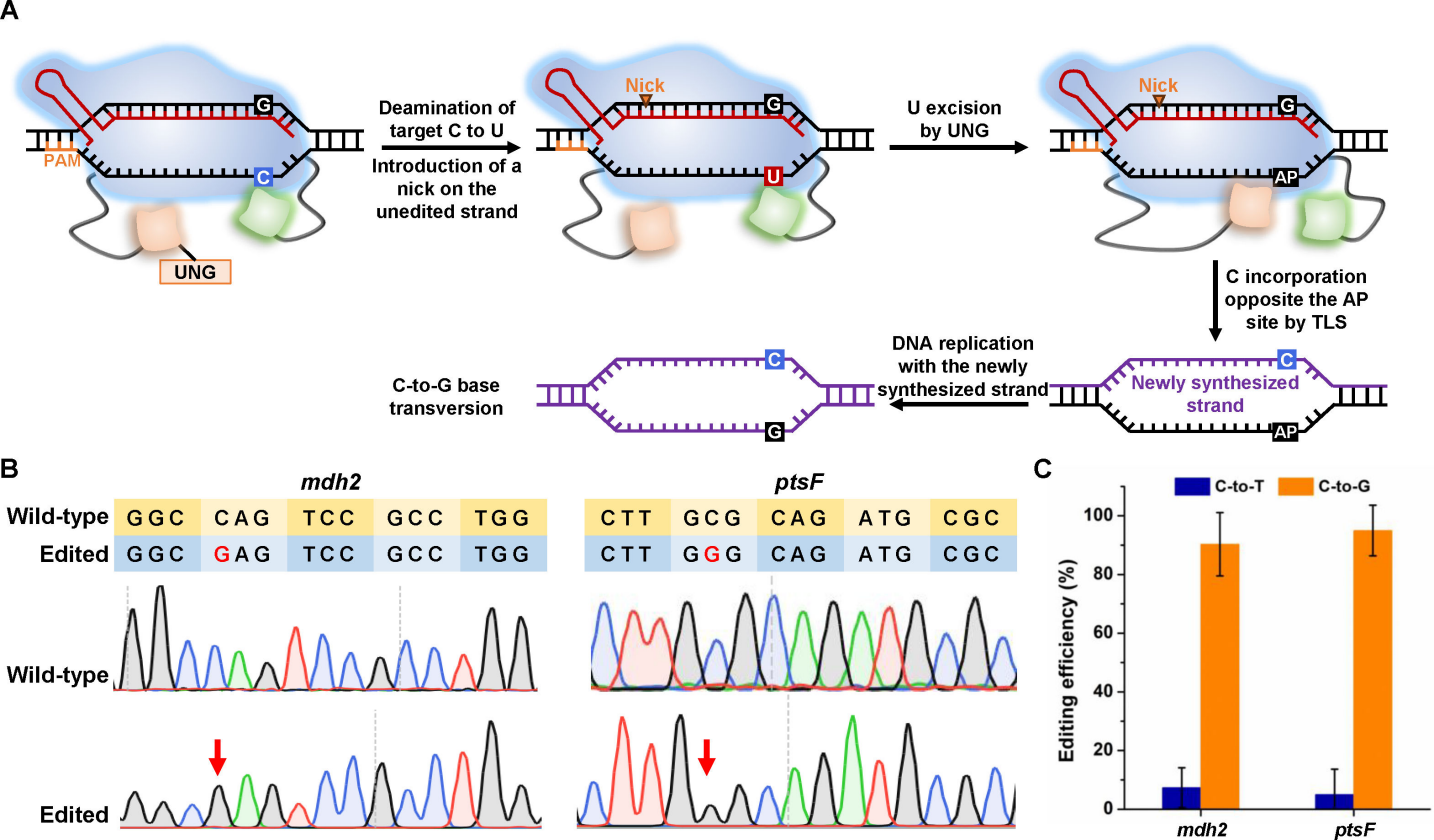
C-to-G base editing by UNG-nCas9(D10A)-AID in *K. xylinus*. (**A**) Schematic representation of the mechanism of C-to-G base editing by UNG-nCas9(D10A)-AID fusion guided by gRNA. C on the target strand is deaminated to U by AID. Meanwhile, a nick is introduced into the non-target strand by nCas9(D10A). U is excised by UNG to form an AP site. The edited strand carrying the AP site is used as the template for DNA replication and C is incorporated opposite to the AP site by TLS enzymes. Subsequent DNA replication with the newly synthesized strand as the template results in C-to-G conversion. (**B**) Analysis of the wild type and the UNG-nCas9(D10A)-AID edited strain by Sanger sequencing. The yellow and blue lines represent the wild type and edited sequences. The G in red represents the edited nucleotide. (**C**) Base editing efficiency with UNG-nCas9(D10A)-AID. Error bars indicate standard deviations from three parallel experiments.

The cytosine base editor nCas9(D10A)-AID efficiently introduces a C-to-T base transition in *K. xylinus*. A glycosylase base editor (GBE) comprising a Cas9 nickase, cytidine deaminase, and UNG has been developed to introduce C-to-A transversions in *Escherichia coli* and C-to-G transversions in mammalian cells (15, 16). To expand the base editing type of the *K. xylinus* base editor, a GBE was developed by fusing a UNG to the N-terminal of the cytosine base editor nCas9(D10A)-AID (Fig. 4A). Interestingly, C-to-G transversions were the main products of GBE in *K. xylinus*, which was consistent with that observed in mammalian cells but different from that observed in *E. coli* (Fig. 4B). For the two tested *K. xylinus* target genes, the C-to-G base editing efficiencies were both >90% with <10% C-to-T conversion (Fig. 4C).

Although both *K. xylinus* and *E. coli* are Gram-negative bacteria, the outcomes of GBE editing are different (15). In a previous study on the Gram-positive bacterium *C. glutamicum*, C-to-G was also the main product of GBE (31). TLS DNA polymerases with substrate specificity were reported to control the outcome of GBE in different microorganisms (31, 32). However, whether the C-to-A transversion in *E. coli* is an exception remains inconclusive. Testing GBE in more microorganisms and systematically analyzing the substrate specificity of TLS DNA polymerases may decipher the rule behind the product preference of GBE in different organisms.

### Application of the base editing tool in other *Komagataeibacter* species

**Fig 5:**
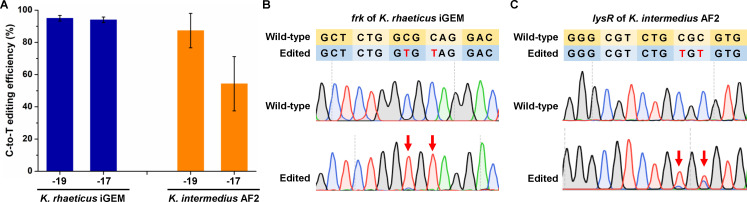
Application of the developed base editing tool in other *Komagataeibacter* species. (**A**) Base editing efficiency in *frk* of *K. rhaeticus* iGEM and *lysR* of *K. intermedius* AF2. Error bars indicate standard deviations from three parallel experiments. (**B**) Representative Sanger sequencing peaks for base editing in *frk* of *K. rhaeticus* iGEM. The yellow and blue lines represent the wild type and edited sequences. The Ts in red and the red arrows represent the edited nucleotides. (**C**) Representative Sanger sequencing peaks for base editing in *lysR* of *K. intermedius* AF2. The yellow and blue lines represent the wild type and edited sequences. The Ts in red and the red arrows represent the edited nucleotides.

The application of the developed base editing tool was then tested in two other *Komagataeibacter* species, *K. rhaeticus* iGEM and *K. intermedius* AF2. Sequence blast suggested that gRNA_frk_ designed for *K. xylinus* CGMCC 2955 could be directly used for targeting the *frk* gene in *K. rhaeticus* iGEM. Therefore, the cytosine base editor nCas9(D10A)-AID-gRNA_frk_ plasmid was electrotransformed into *K. rhaeticus* iGEM. Transformants were randomly selected to detect base editing efficiency by Sanger sequencing the target region. Similar to *K. xylinus* CGMCC 2955, high-efficiency C-to-T base editing was observed in *K. rhaeticus* iGEM even without IPTG induction. The C-to-T base editing efficiency was 95% (Fig. 5). For *K. intermedius* AF2, we did not find the same sequences that could be targeted by gRNAs designed for *K. xylinus* CGMCC 2955. Therefore, we designed a gRNA_lysR_ targeting the LysR family transcriptional regulator GLUCOINTEAF2_0203748 encoding gene of *K. intermedius* AF2. Using the same cytosine base editor, nCas9(D10A)-AID, C-to-T base editing was achieved with an efficiency of 87% (Fig. 5). These results suggest the applicability of the developed base editing tool in various *Komagataeibacter* species.

### Application of base editing in identifying mannitol metabolism-related genes

The sugar alcohol mannitol is a major component of brown seaweeds. Owing to its higher energy density than glucose and wide availability, mannitol has become a promising feedstock for biomanufacturing (25, 33). Some *K. xylinus* strains have been reported to metabolize mannitol for BC production (34). However, the mannitol metabolism-related genes are largely unknown. Because base editing is an efficient tool for gene inactivation, we used base editing technology to investigate the mannitol metabolism-related genes in *K. xylinus* CGMCC 2955.

**Fig 6:**
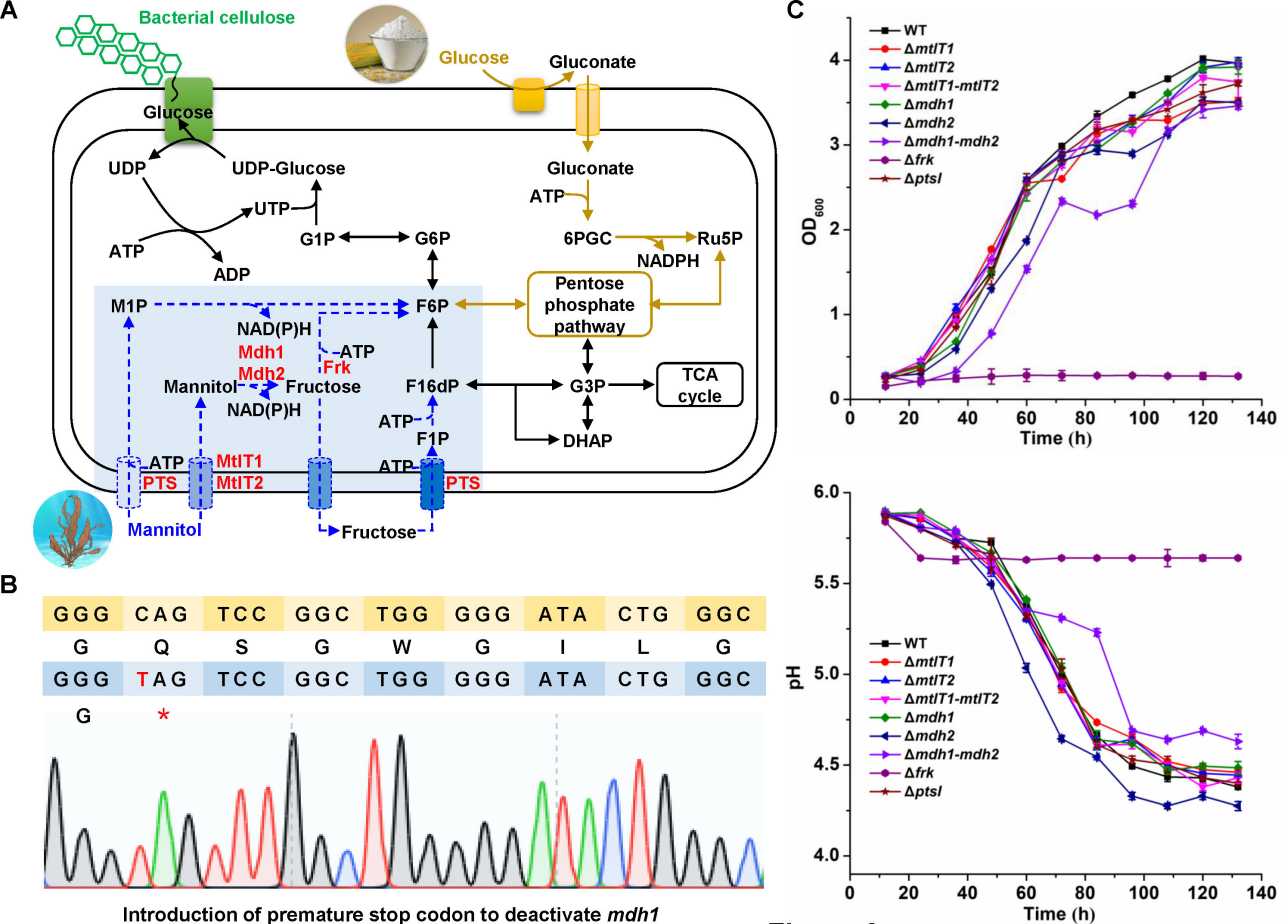
Investigation of mannitol metabolism-related genes in *K. xylinus* CGMCC 2955 using base editing. (**A**) Metabolic pathways for glucose and mannitol catabolism and BC biosynthesis. The predicted mannitol metabolic pathways are indicated by dotted blue arrows. The predicted enzymes involved in mannitol metabolism are indicated in red. (**B**) Introduction of a premature stop codon in *mdh1*. The yellow and blue lines represent the wild type and edited sequences. The T in red represents the edited nucleotide. The star in red represents translation termination. The results for deactivating other mannitol metabolic genes are shown in Fig. S1. (**C**) Cell growth on mannitol and pH change of the fermentation broth. Error bars indicate standard deviations from three parallel experiments.

Microorganisms mainly use two metabolic pathways to convert mannitol into the glycolysis intermediate fructose-6-phosphate: phosphorylation-dehydrogenation and dehydrogenation-phosphorylation (Fig. 6A) (25). A genomic analysis of *K. xylinus* CGMCC 2955 suggests the existence of two MFS transporters for mannitol uptake (encoded by *mtlT1* and *mtlT2*), two mannitol 2-dehydrogenase for oxidation of mannitol to fructose (encoded by *mdh1* and *mdh2*), and a fructokinase (encoded by *frk*). Furthermore, *K. xylinus* CGMCC 2955 has been reported to not possess a functional phosphotransferase system (PTS) for glucose uptake. However, several PTS genes were annotated in its genome. These genes have sequence similarities with their homologs in both *E. coli* and *C. glutamicum* (Table 1).

**TABLE 1:** Predicted mannitol metabolic genes of *K. xylinus* CGMCC 2955

Gene	Product	AA length	Identity with the *E. coli* homolog	Identity with the *C. glutamicum* homolog
*mtlT1*	MFS transporter	438	–^*a*^	39%
*mtlT2*	MFS transporter	434	–	39%
*mdh1*	Mannitol 2-dehydrogenase	493	–	39%
*mdh2*	Mannitol 2-dehydrogenase	487	–	41%
*frk*	Fructokinase	302	49%	–
*ptsF*	Fructose transporter (PTS subunit IIA)	153	28%	–
*ptsH*	Phosphocarrier protein (PTS subunit HPr)	98	–	–
*ptsI*	Phosphoenolpyruvate-protein phosphotransferase (PTS subunit I)	612	33%	32%

^*a*^ –, no sequence identity.

Next, these genes were deactivated by the base-editing-mediated introduction of premature stop codons (Fig. 6B; Fig. S6). Gene-deactivated mutants were subjected to growth tests using mannitol as the sole carbon source. Unexpectedly, single or double inactivation of the predicted mannitol transporter genes, *mtlT1,* and *mtlT2* did not cause significant changes in cell growth on mannitol, excluding their roles in mannitol uptake (Fig. 6C). Inactivation of *ptsI* had no obvious effect on mannitol metabolism. This observation is consistent with previous findings showing that the PTS system is not functional in *K. xylinus* CGMCC 2955. Single inactivation of *mdh1* or *mdh2* slightly slowed cell growth. Combinational inactivation of *mdh1* and *mdh2* further slowed cell growth on mannitol but did not completely abolish mannitol metabolism (Fig. 6C). The results suggest that although *mdh1* and *mdh2* participate in mannitol oxidation, other unknown isoenzymes exist in *K. xylinus* CGMCC 2955. The sequence-based classification suggests that Mdh belongs to the family of polyol-specific long-chain dehydrogenases/reductases (35). According to the genome annotation, *K. xylinus* CGMCC 2955 has many dehydrogenases, including sugar (phosphate) dehydrogenases and glycerol (phosphate) dehydrogenases. Their function in catalyzing mannitol oxidation can be investigated in future research. Only inactivation of *frk* completely abolished cell growth on mannitol, proving that mannitol was firstly oxidized to fructose and then phosphorylated by fructokinase. The medium also did not acidify for the *frk*-deactivated mutant (Fig. 6C). Frk of *K. xylinus* CGMCC 2955 has a 49% amino acid sequence identity with the *E. coli* homolog. Inactivation of *frk* also completely abolished cell growth in the minimal medium with fructose as the sole carbon source (Fig. S7).

In summary, the dehydrogenation-phosphorylation pathway is the only functional mannitol metabolic pathway in *K. xylinus* CGMCC 2955, whereas key genes involved in non-PTS mannitol transport and dehydrogenation remain unclear. However, it is difficult to identify these genes if they share little sequence similarity with the known ones. For future research, comparable transcriptomic analyses for strains cultivated using glucose and mannitol as carbon sources may help find mannitol-induced genes. Further inactivation of these genes using the developed base editing technology can be conducted to examine their function in mannitol metabolism.

### Conclusions

In this study, to address the challenge of efficient and multiplex genome editing in BC-producing *Komagataeibacter*, we developed CRISPR-guided base editing technologies that work in various *Komagataeibacter* species. C-to-T and C-to-G editing were efficiently introduced into chromosomal targets using different base editors. Single-, double-, and triple-gene editing with >80% efficiency was achieved. The developed base editing tools were used to investigate the mannitol metabolism in *K. xylinus*. This study provides a useful technology for engineering *Komagataeibacter* to enhance BC production from renewable feedstocks.

## MATERIALS AND METHODS

### Strains and culture conditions

The bacterial strains used in this study are listed in Table S3. Notably, *E. coli* strains DH5α and DB3.1 were used for general cloning and cultured aerobically at 37°C in Luria–Bertani (LB) broth. Chloramphenicol (Cm, 20 µg/mL) was added to LB broth as required. *Komagataeibacter* strains (*K. xylinus* CMCC2955, *K. intermedius* AF2, and *K. rhaeticus* iGEM) were cultured at 30°C in HS medium (0.75% yeast extract, 1% peptone, 1% Na_2_HPO_4_, and 2.5% glucose; pH 6.0). Cm (340 µg/mL) and different concentrations of IPTG were added to the medium as required. For mannitol fermentation, MA/9 medium (0.552% Na_2_HPO_4_·2H_2_O, 0.34% KH_2_PO_4_, 0.1% NH_4_Cl, 0.0008% nitrilotriacetic acid, 0.1% NaCl, 0.025% MgSO_4_·7H_2_O, 0.002% CaCl_2_·2H_2_O, 0.0001% FeCl_3_, and 0.02% casein amino acids) was used with 1% mannitol as the carbon source.

### Plasmid construction and gRNA design

The plasmids and primers used in this study are listed in Tables S3 and S4, respectively. The gRNA expression cassette consisted of a promoter P*_tac_*, a 20 bp guide sequence (complementary region for specific DNA binding), and a hairpin for Cas9 binding. The gRNAs used for gene activation were designed using gBIG (http://gbig.ibiodesign.net/) (27) and are listed in Table S5. To generate the plasmids pdCas9-AID and pnCas9(D10A)-AID, encoding genes for dCas9-AID and nCas9(D10A)-AID and gRNA expression cassette were cloned to the pSEVA331 plasmid under the control of IPTG inducible promoter P*_tac_* (26). To improve the efficiency of constructing gRNA expression plasmids, the 20 bp guide sequence region in the plasmid was replaced by a *Bsa*I-*ccdB-Bsa*I cassette, generating plasmids pdCas9-AID-gRNA and pnCas9(D10A)-AID-gRNA in *E. coli* DB3.1. For constructing plasmid pUNG-nCas9(D10A)-AID-gRNA, the UNG-linker fragment was amplified from the plasmid pUNG_Ec_-nCas9(D10A)-AID-gRNA-*ccdB*^TS^ by PCR (31). The linearized fragment of the plasmid pnCas9(D10A)-AID-gRNA was amplified by PCR. The two fragments were ligated using a ClonExpress MultiS One Step Cloning Kit (Vazyme, Nanjing, China) to construct pUNG-nCas9(D10A)-AID-gRNA. A Golden Gate assembly strategy was used to facilitate the rapid and simple construction of gRNA expression plasmids. First, a pair of 24 bp primers was annealed to form double-stranded DNA with cohesive ends. Next, the double-stranded DNA was ligated to pdCas9-AID-gRNA, pnCas9(D10A)-AID-gRNA, or pUNG-nCas9(D10A)-AID-gRNA using the Golden Gate assembly reaction to generate a gRNA expression plasmid with a guide sequence.

### Base editing procedure

Base editing plasmid expressing targeting gRNA (approximately 2 µg) was transformed into *Komagataeibacter* strains via electroporation. The cells were incubated at 30°C for 12 h and then spread onto HS plates supplemented with Cm. The plates were incubated at 30°C for 48–72 h until colonies appeared. Colonies were selected and cultured on HS plates supplemented with Cm. For IPTG induction, the colonies were transferred to fresh HS medium supplemented with Cm and IPTG with an initial OD_600_ of 0.05 to induce the expression of the base editor. After 24 h of culture, the cells were washed twice and resuspended in a sterilized 0.85% NaCl solution. The cells were collected and used to analyze the base editing efficiency. The target region was amplified by PCR to verify the editing events by Sanger sequencing. The primers used for PCR amplification and Sanger sequencing are listed in Table S4. The Sanger sequencing data were aligned to the reference sequence. The base editing efficiency was analyzed by calculating the occurrence frequency of each base at each position of the target region using EditR 1.0.10 (https://moriaritylab.shinyapps.io/editr_v10/) (36). The C-to-T editing efficiency was defined as the occurrence frequency of T/(A + T + C + G). The C-to-G editing efficiency was defined as the occurrence frequency of G/(A + T + C + G).

### Gene inactivation by base editing

Base editing-mediated introduction of a premature stop codon was used for gene inactivation in *K. xylinus* CMCC 2955. All gRNA sequences of *K. xylinus* CMCC 2955 were identified and analyzed using the bioinformatics tool gBIG (http://gbig.ibiodesign.net/) (27). CRISPR/nCas9(D10A)-AID targeting CGA (Arg), CAG (Gln), and CAA (Gln) on the coding strand can create TGA (opal), TAG (amber), and TAA (ochre) stop codons via C-to-T editing. Additionally, CRISPR/nCas9(D10A)-AID targeting CCA in the non-coding strand can create TGA, TAG, and TAA stop codons in the coding strand by changing either or both C to T.

### Plasmid curing

To obtain plasmid-free edited strains for subsequent growth tests, edited *K. xylinus* CMCC 2955 strains containing plasmids were incubated overnight at 30°C in HS medium without Cm. The cultures were diluted with a sterilized 0.85% NaCl solution and spread onto HS plates with or without Cm. After incubation at 30°C for 48 h, the plasmid curing rate was calculated by counting colonies formed. Colonies grown on the HS plates without Cm were selected to test their sensitivity to Cm. Those sensitive to Cm were edited strains that had successfully undergone plasmid curing. The plasmid curing efficiency was defined as the number of colonies sensitive to Cm divided by the number of all the tested colonies.

### Mannitol fermentation of *K. xylinus*

Plasmid-cured edited strains and the wild-type control strain were inoculated into shake flasks with HS medium and cultured at 30°C with shaking at 180 rpm. When the OD_600_ reached 0.5–0.6, the cells were collected by centrifugation and washed twice with a sterilized 0.85% NaCl solution. Subsequently, cells were inoculated into 75 mL fresh MA/9 media in 250 mL flasks with an initial OD_600_ of 0.02 and cultured at 30°C with shaking at 180 rpm. The pH of the medium was recorded every 24 h using a PHSJ-4A basic benchtop pH meter (Leici Corporation, China) and cell density was monitored by measuring the OD_600_ with a UV−Vis spectrophotometer (UV-6100, Mapada, China).

### Whole genome resequencing

The genomic DNAs of base-edited *K. xylinus* strains were extracted using the Promega Wizard Genomic DNA Purification Kit (Madison, WI, USA). Library construction, whole genome resequencing, and analysis of off-target mutations were conducted by MajorBio (Shanghai, China). Specifically, NGS was performed using the Illumina Hiseq2500 sequencing platform. The output was analyzed for quality assurance by using FastQC software (v.0.10.1). The MUMmer software (version 3.23) (37) was used to align the genome of the edited strain and the reference genome of the wild-type strain using the parameters -maxmatch -c 65 -l 20 and then used the delta -filter -m parameter with the many-to-many alignment block option to filter the alignment results. The snpEff software (v.4.3i) (38) was used to annotate the SNPs. The genome of the wide-type strain was used as a reference for identifying the off-target mutations in the edited strains.

## Data Availability

The data supporting the findings of this work are available within the paper and the supplemental material files. The data sets generated and analyzed during this study are available from the corresponding author upon request. The genome resequencing data have been uploaded to the NCBI database under accession number PRJNA1189666. The wild-type *K. xylinus* strain CGMCC 2955 used in this study has been sequenced by our group, and the genome sequence has been uploaded to GenBank under accession number CP024644.1.

## SUPPLEMENTAL MATERIAL

Supplemental material aem.02455-24-s0001.docx Figures S1 to S7; Tables S1 and S3 to S5.

Table S2 aem.02455-24-s0002.xlsx Genome-scale analysis of gRNAs for base editing-mediated introduction of premature stop codon.

ASM does not own the copyrights to Supplemental Material that may be linked to, or accessed through, an article. The authors have granted ASM a non-exclusive, world-wide license to publish the Supplemental Material files. Please contact the corresponding author directly for reuse.
