# Supplementary material for: CRISPR-guided base editor enables efficient and multiplex genome editing in bacterial cellulose-producing Komagataeibacter species
Source: Appl Environ Microbiol. 2025 Jan 31;91(2):e02455-24. doi: 10.1128/aem.02455-24 (PMC11837512; doi:10.1128/aem.02455-24)
Supplement: Supplemental material — Figures S1 to S7; Tables S1 and S3 to S5. [file aem.02455-24-s0001.docx]

**Supplementary material**

**
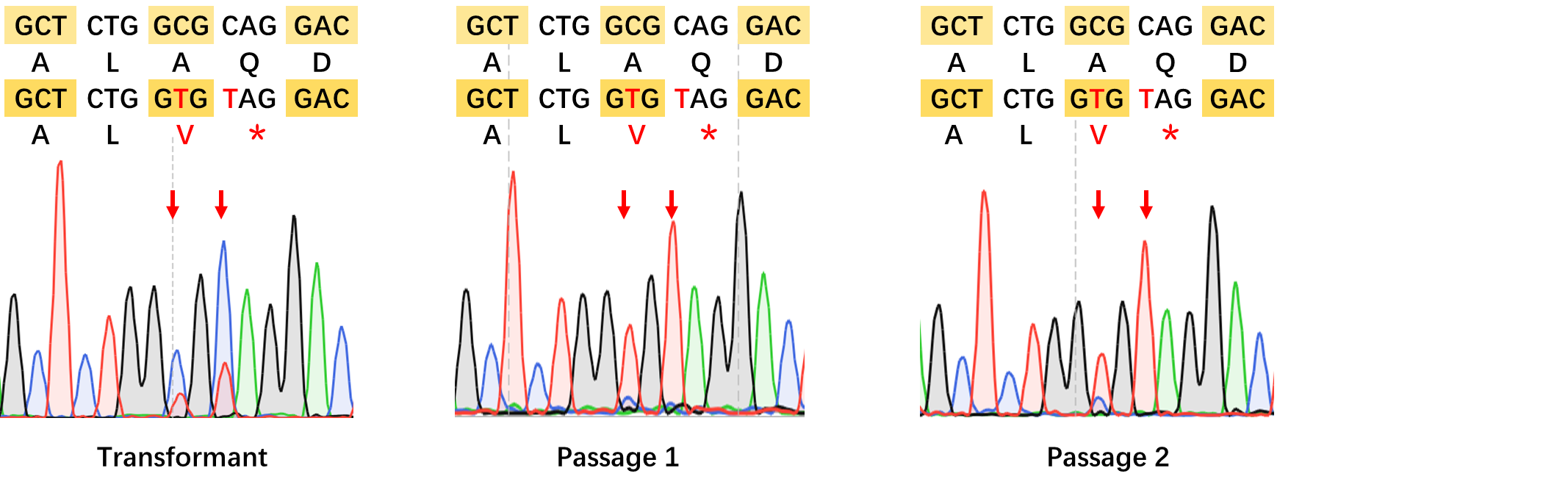
**

**Figure S1.** Representative Sanger sequencing peaks for base editing in the transformant, passage 1, and passage 2.

**
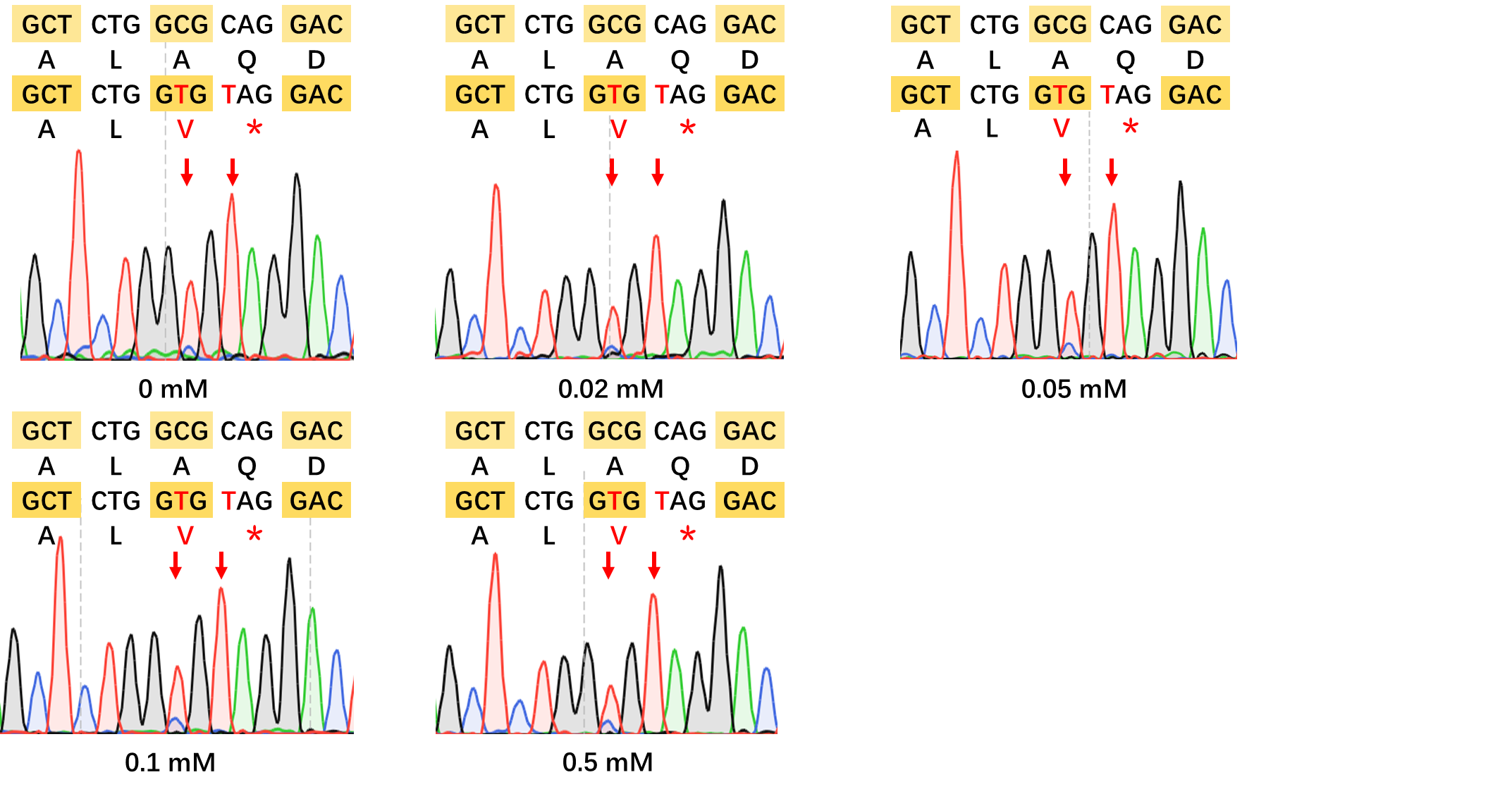
**

**Figure S2.** Representative Sanger sequencing peaks for base editing with different IPTG usages.


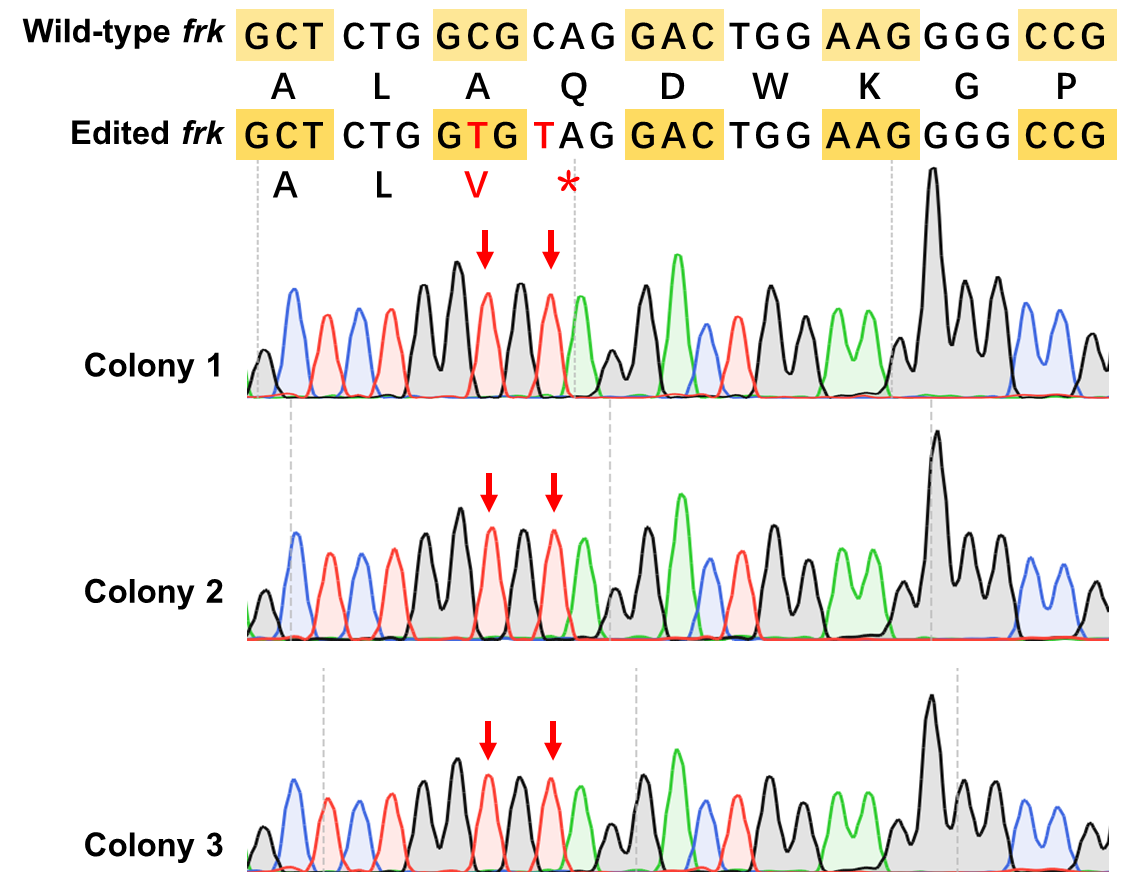


**Figure S3.** Sanger sequencing peaks for three base edited colonies that were isolated from the culture of plasmid curing. The results suggest the fully base editing at the target *frk* locus after base editing and plasmid curing.

**
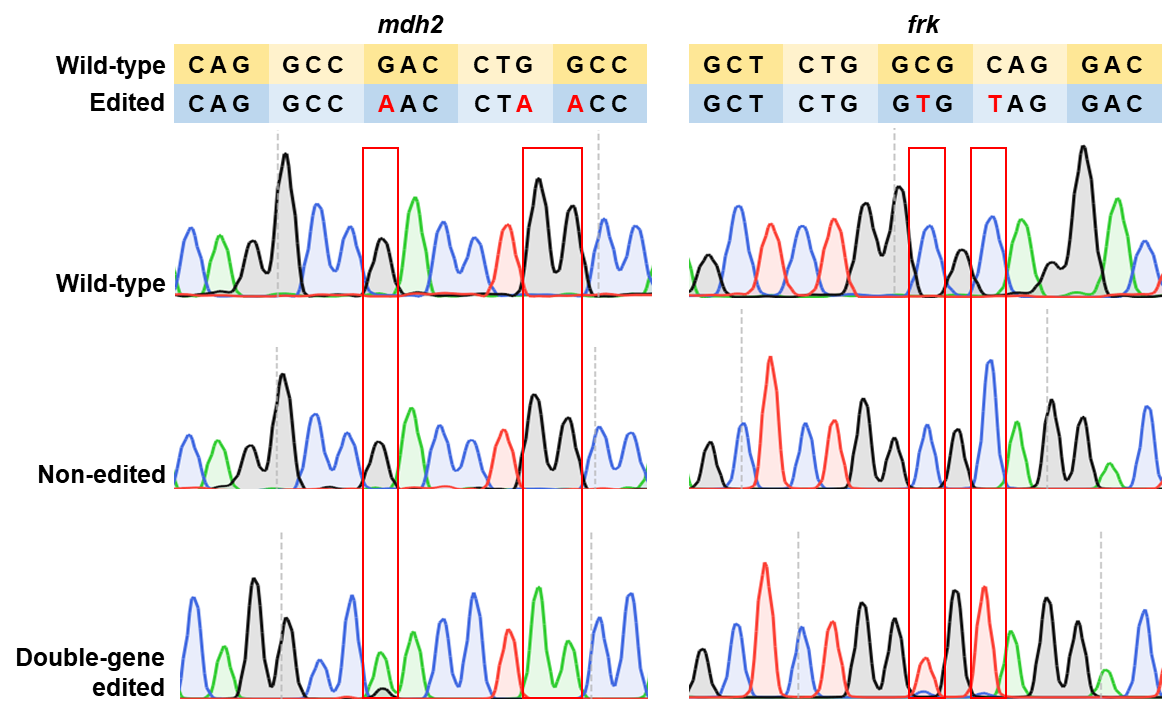
**

**Figure S4.** Representative Sanger sequencing peaks for the double-gene editing colonies.


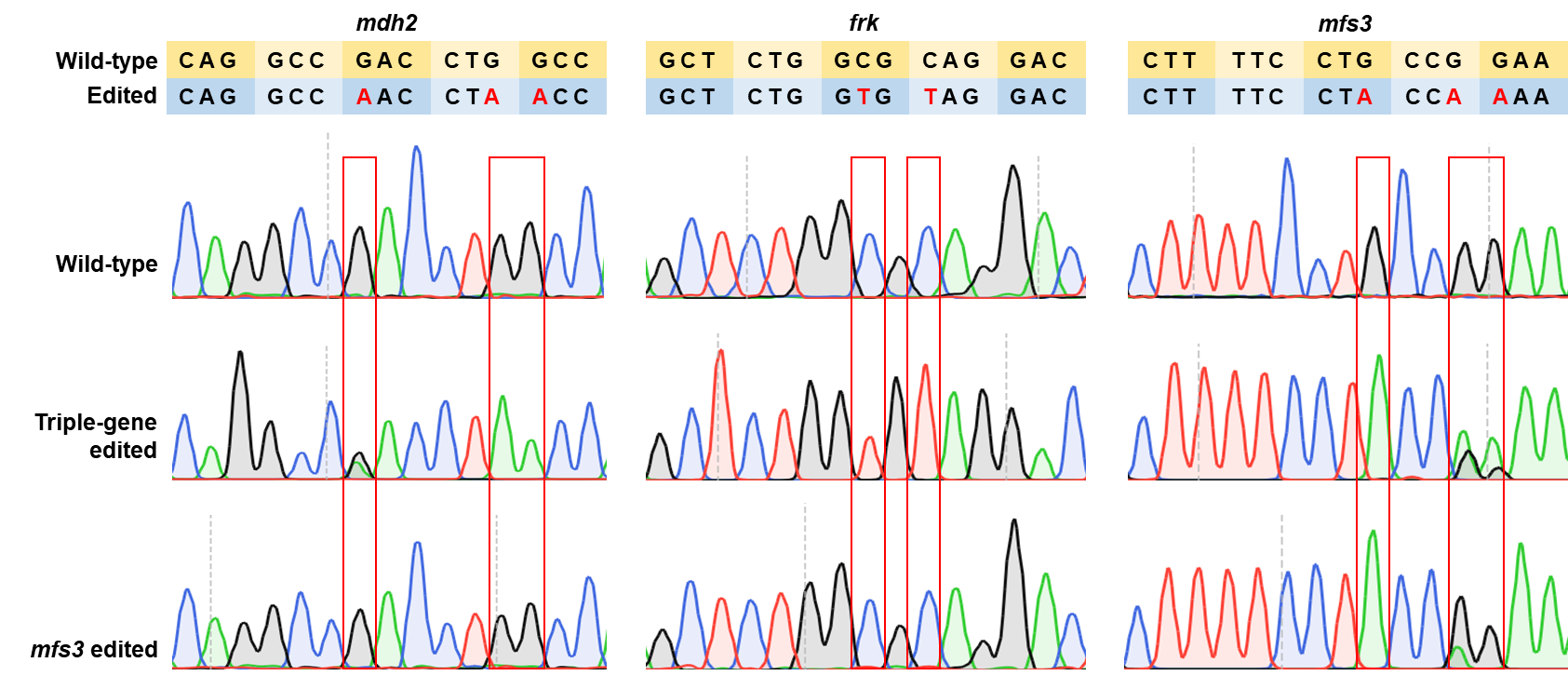


**Figure S5.** Representative Sanger sequencing peaks for the triple-gene editing colonies.

**
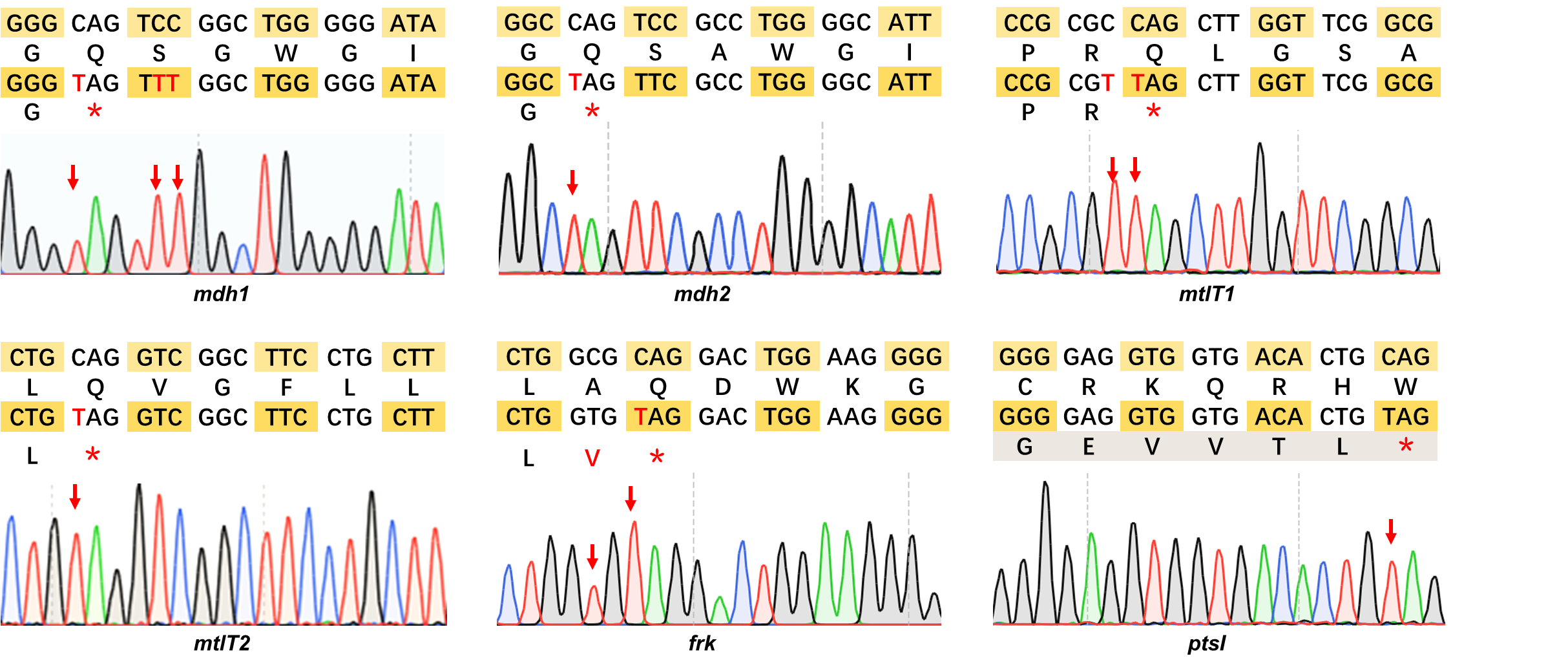
**

**Figure S6.** Introduction of premature stop codons in mannitol metabolic genes by C-to-T base editing. Colonies after base editing and plasmid curing were tested for the generation of premature stop codons.


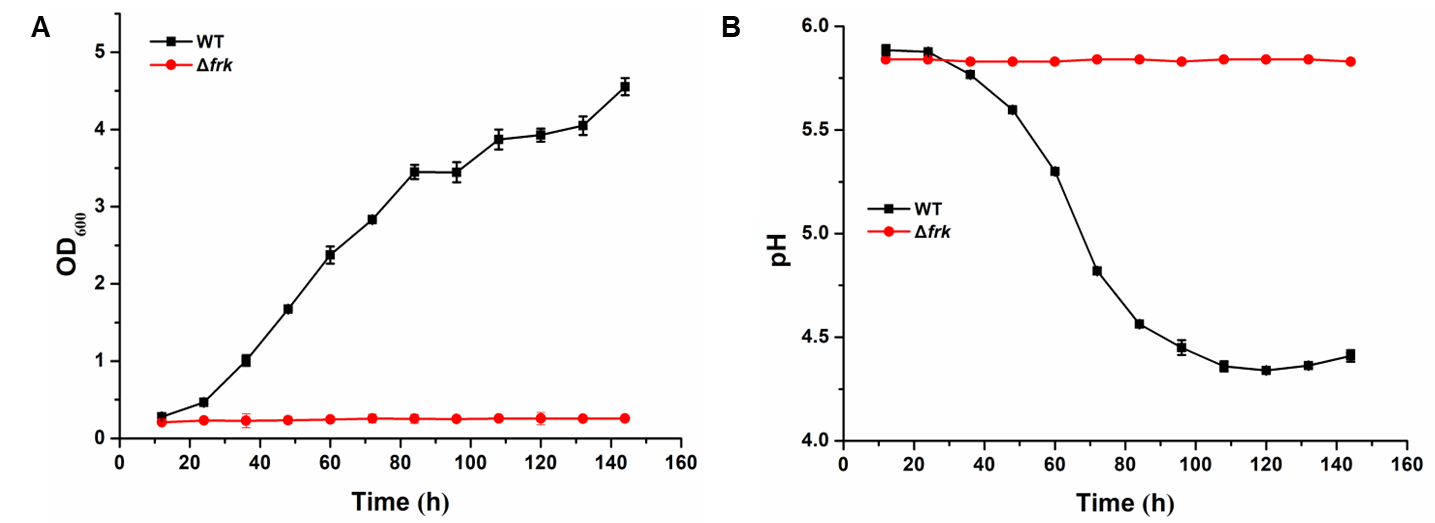


**Figure S7.** Growth of the wild-type and *frk*-deactivated *K. xylinus* CGMCC 2955 strains in the minimal medium with fructose as the sole carbon source. (A) Cell growth. (B) pH changes of the medium. Error bars indicate standard deviations from three parallel experiments.

**Table S1.** Analysis of SNPs in the edited strains by genome resequencing. The targeted base conversion by the base editor is highlighted in red.

|  | **Position** | **Reference** | **Alteration** |
| --- | --- | --- | --- |
| ***mdh2*-edited strain 1** | 481743 | C | T |
|  | 2103503 | G | A |
|  | 2295490 | GG | AA |
|  | 2941921 | C | T |
|  | 3154552 | G | A |
| ***mdh2*-edited strain 2** | 3154552 | G | A |
| ***mdh2*-edited strain 3** | 481743 | C | T |
|  | 1701606 | C | T |
|  | 2185130 | G | A |
|  | 2630042 | C | T |
|  | 3154526 | C | T |
|  | 3154552 | G | A |
|  | 3362926 | G | A |
| ***mtlT1*-edited strain 1** | 207166 | C | T |
|  | 2196506 | G | A |
|  | 2745139 | G | A |
|  | 2835763 | G | A |
|  | 3146800 | G | A |
|  | 3395394 | G | A |
| ***mtlT1*-edited strain 2** | 2359979 | G | A |
|  | 2912070 | C | T |
|  | 3146800 | G | A |
|  | 3196759 | G | A |
| ***mtlT1*-edited strain 3** | 3311075 | CCAGC | TTAGT |
|  | 1469819 | G | A |
|  | 2659581 | C | T |
|  | 3146800 | G | A |

**Table S3.** Strains and plasmids used in this study.

| **Strain or plasmid** | **Description^a^** | **Reference or source** |
| --- | --- | --- |
| **Strain** |  |  |
| *E. coli* DH5α | General cloning host | TaKaRa |
| *E. coli* DB3.1 | Cloning host for ccdB containing plasmids | TransGen  Biotech |
| *K. xylinus* CMCC 2955 | Wild-type strain | Lab stock |
| *K. intermedius* AF2 | Wild-type strain | Lab stock |
| *K. rhaeticus* iGEM | Wild-type strain | HZBio，Microbial Conservation Platform |
| Δ*mdh1* | CGMCC 2955 derivative with its *mdh1* gene deleted | This study |
| Δ*mdh2* | CGMCC 2955 derivative with its *mdh2* gene deleted | This study |
| Δ*mdh1-mdh2* | CGMCC 2955 derivative with its *mdh1* and *mdh2* genes deleted | This study |
| Δ*mtlT1* | CGMCC 2955 derivative with its *mtlT1* gene deleted | This study |
| Δ*mtlT2* | CGMCC 2955 derivative with its *mtlT2* gene deleted | This study |
| Δ*mtlT1*-Δ*mtlT2* | CGMCC 2955 derivative with its *mtlT1* and *mtlT2* genes deleted | This study |
| Δ*frk* | CGMCC 2955 derivative with its *frk* gene deleted | This study |
| Δ*ptsI* | CGMCC 2955 derivative with its *ptsI* gene deleted | This study |
| **Plasmid^b^** |  |  |
| pSEVA331 | pBBR1 ori, CmR | [^1^](#_ENREF_1) |
| pnCas9(D10A)-AID-gRNA-*ccdB*^TS^ | pXMJ19 derivative harboring nCas9(D10A)-AID and gRNA expression cassettes, temperature sensitive | [^2^](#_ENREF_2) |
| pUNG_Ec_-nCas9(D10A)-AID-gRNA-*ccdB*^TS^ | pnCas9(D10A)-AID-gRNA-*ccdB*^TS^ derivative with *ung* gene from *E. coli* fused to the N-terminal of nCas9(D10A)-AID | [^3^](#_ENREF_3) |
| pdCas9-AID | pSEVA331 derivative, carrying dCas9-AID cassette driven by IPTG-inducible promoter Ptac | This study |
| pnCas9(D10A)-AID | pSEVA331 derivative, carrying nCas9(D10A)-AID cassette driven by IPTG-inducible promoter Ptac | This study |
| pdCas9-AID-gRNA | pdCas9-AID derivative, the 20-bp guide sequence is replaced with a *Bsa*I-*ccdB*-*Bsa*I cassette to facilitate easy assembly of guide sequence | This study |
| pnCas9(D10A)-AID-gRNA | pnCAS9-AID derivative, the 20-bp guide sequence is replaced with a *Bsa*I-*ccdB*-*Bsa*I cassette to facilitate easy assembly of guide sequence | This study |
| pUNG-nCas9(D10A)-AID-gRNA | pnCas9(D10A)-AID-gRNA derivative, the uracil-DNA glycosylase (UNG) from *E. coli* was fused at the N-terminal of nCas9(D10A) | This study |
| pnCas9(D10A)-AID*-frk* | pnCas9(D10A)-AID-gRNA derivative, carrying the gRNA cassette for *frk* editing in *K. xylinus* CMCC 2955 and *K. rhaeticus* iGEM | This study |
| pnCas9(D10A)-AID*-mdh1* | pnCas9(D10A)-AID-gRNA derivative, carrying the gRNA cassette for *mdh1* editing | This study |
| pnCas9(D10A)-AID*-mdh2-1* | pnCas9(D10A)-AID-gRNA derivative, carrying the gRNA-1 cassette for *mdh2* editing | This study |
| pnCas9(D10A)-AID*-mdh2-2* | pnCas9(D10A)-AID-gRNA derivative, carrying the gRNA-2 cassette for *mdh2* editing | This study |
| pnCas9(D10A)-AID*-mdh2-3* | pnCas9(D10A)-AID-gRNA derivative, carrying the gRNA-3 cassette for *mdh2* editing | This study |
| pnCas9(D10A)-AID-*mdh2-4* | pnCas9(D10A)-AID-gRNA derivative, carrying the gRNA-4 cassette for *mdh2* editing | This study |
| pnCas9(D10A)-AID-*mdh2-5* | pnCas9(D10A)-AID-gRNA derivative, carrying the gRNA-5 cassette for *mdh2* editing | This study |
| pnCas9(D10A)-AID-*mdh2* | pnCas9(D10A)-AID-gRNA derivative, carrying the gRNA cassette for deactivating *mdh2* | This study |
| pnCas9(D10A)-AID*-ptsF* | pnCas9(D10A)-AID-gRNA derivative, carrying the gRNA cassette for *ptsF* editing | This study |
| pnCas9(D10A)-AID*-ptsI* | pnCas9(D10A)-AID-gRNA derivative, carrying the gRNA cassette for *ptsI* editing | This study |
| pnCas9(D10A)-AID*-mtlT1* | pnCas9(D10A)-AID-gRNA derivative, carrying the gRNA cassette for *mtlT1* editing | This study |
| pnCas9(D10A)-AID*-mtlT2* | pnCas9(D10A)-AID-gRNA derivative, carrying the gRNA cassette for *mtlT2* editing | This study |
| pnCas9(D10A)-AID*-mtlT1-2* | pnCas9(D10A)-AID-gRNA derivative, carrying the gRNA cassette for *mtlT1* and *mtlT2* editing | This study |
| pnCas9(D10A)-AID*-mfs3* | pnCas9(D10A)-AID-gRNA derivative, carrying the gRNA cassette for *mfs3* editing | This study |
| pnCas9(D10A)-AID*-mdh1-2* | pnCas9(D10A)-AID-gRNA derivative, carrying the gRNA cassettes for *mdh1* and *mdh2* editing | This study |
| pnCas9(D10A)-AID*-mdh2-fk* | pnCas9(D10A)-AID-gRNA derivative, carrying the gRNA cassettes for *mdh2* and *frk* editing | This study |
| pnCas9(D10A)-AID*-mdh2-fk-mfs3* | pnCas9(D10A)-AID-gRNA derivative, carrying the gRNA cassettes for *mdh2*, *frk* and *mfs3* editing | This study |
| pUNG-nCas9(D10A)*-mdh2* | pUNG-nCas9(D10A)-AID-*ccdB* derivative, carrying the gRNA cassette for *mdh2* editing | This study |
| pUNG-nCas9(D10A)*-ptsF* | pUNG-nCas9(D10A)-AID-*ccdB* derivative, carrying the gRNA cassette for *ptsF* editing | This study |
| pnCas9(D10A)-AID*-lysR* | pnCas9(D10A)-AID-gRNA derivative, carrying the gRNA cassette for *lysR* editing in *K. intermedius* AF2 | This study |

^a^Cm^R^ represents resistance to chloramphenicol. A full list of guide sequences used in this study is shown in Table S5.

**Table S4.** Primers used in this study.

| **Primers** | **Sequence (5’-3’)** | **Relevance** |
| --- | --- | --- |
| cm-F | acctgcatcgatttaaaacagacgaagaatccatggg | Reverse PCR to prepare linearized pSEVA331 vector |
| cm-R | TCCATCTAGTATTTCTCCTCTTTCACTAGttgtg |  |
| cas9-F | GAGGAGAAATACTAGatggataagaaatactcaataggcttagctatcggcacaaatagcgtcgg | PCR amplification of the nCas9 region |
| nAid-2-R | aactttgtggaacaatgtgatcg |  |
| nAid-3-F | tcacattgttccacaaagtttccttaaagacgattcaatagac | PCR amplification of the nCas9, linker and AID region |
| cas9-R | aacaggagtccaagCttaaccagagccgcgggacacgg |  |
| grna-F | gttaaGcttggactcctgttgatag | PCR amplification of the gRNA cassette region |
| grna-R | tgttttaaatcgatgcaggtggcac |  |
| cas9-F | GAGGAGAAATACTAGatggataagaaatactcaataggcttagctatcggcacaaatagcgtcgg | PCR amplification of the dCas9 region |
| dAid-2-R | AACTTTGTGGAACAATGGCATCG |  |
| dAid-3-F | tgccattgttccacaaagttTCCTTAAAGACGATTCAATAGAC | PCR amplification of the dCas9, linker and AID region |
| cas9-R | aacaggagtccaagCttaaccagagccgcgggacacgg |  |
| ccdb-U | CTCGAGcATCGATGCacgcgtggatccggcttact | PCR amplification of the *ccdB* gene |
| ccdb-D | CGGCCttatattccccagaacatcaggttaatggcg |  |
| 419GJ-U | gggaatataaGGCCGCGGTCTCgtgtgtga | Reverse PCR to prepare linearized pnCas9(D10A)/dCas9-AID vector |
| 419GJ-D | cgtGCATCGATgCTCGAGGGTCTCg |  |
| 1211PUng-f | aattgtttgtggagcagcataagcattatttagatgagattattga | Reverse PCR to prepare linearized pnCas9(D10A)-AID-gRNA vector |
| 1211PUng-r | ctagtatttctcctctttcactagttg |  |
| 1211ung-u | tgaaagaggagaaatactagatggctaacgaattaacctgg | PCR amplification of the *ung* and linker region |
| 1211ung-d | atgctgctccacaaacaatt |  |
| P-AIDYZ-D | cctcttacgtgccgatcaac | PCR amplification of the gRNA integrated on plasmids region |
| 221017CX-G | aatgagctggctctgccaag |  |
| MFS1-JYZ-U | AGCGTCACTTCCTCGGCAAT | PCR amplification of the edited *mtlT1* region |
| MFS1-JYZ-D | TTTGCTGACCGGCTGGGCAT |  |
| MFS2-JYZ-U | GTCCAGTTCCGCCTGCAACG | PCR amplification of the edited *mtlT2* region |
| MFS2-JYZ-D | CGGCGATGAAATCCCGCATGA |  |
| MFS3-YZ-U | CGTTGTGATGGTTGATGTGA | PCR amplification of the edited *mfs3* region |
| MFS3-YZ-D | TATTCATTGCGGCATCACTG |  |
| MDH1-JYZ-U | CAATACAGCGTCCCTGCCCC | PCR amplification of the edited *mdh1* gene region |
| MDH1-JYZ-D | ATAGGCCTGCACGAAAGCGG |  |
| MDH2-JYZ-U | CAGTGCGGTCGTGTCCATGT | PCR amplification of the edited *mdh2* gene region |
| MDH2-JYZ-D | GGCGCCACAGTACAGACGGG |  |
| YZIIA-U | GCCGGTCGTGATCATGCATC | PCR amplification of the edited *ptsF* region |
| YZIIA-D | GAACGGTAGGGTGTACCGCT |  |
| YZPTS-U | CCGCCCTGATCGATCCCTC | PCR amplification of the edited *ptsI* region |
| YZPTS-D | CCGCCCTGATCGATCCCTC |  |
| KIL-F | GGCGCTCTTCCCATTTTCATAC | PCR amplification of the edited *lysR* region |
| KIL-R | TGAAGTCGATGAACACCCTGAGC |  |
| FKAID-U | aaacGGCCCCTTCCAGTCCTGCGC | Assembly of the gRNA cassette for *frk* editing in *K. xylinus* and *K. rhaeticus* |
| FKAID-D | cacaGCGCAGGACTGGAAGGGGCC |  |
| 1031mfs1g-U | aacGGCCGCCGAACCAAGCTGGC | Assembly of the gRNA cassette for *mtlT1* editing |
| 1031mfs1g-D | cacaGCCAGCTTGGTTCGGCGGCC |  |
| 1031mfs2g-U | aaacCAAGCAGGAAGCCGACCTGC | Assembly of the gRNA cassette for *mtlT2* editing |
| 1031mfs2g-D | cacaGCAGGTCGGCTTCCTGCTTG |  |
| MFS3-grna-R | cacaCGGCAGGAAAAGCATGCCAA | Assembly of the gRNA cassette for *mfs3* editing |
| MFS3-grna-F | aaacTTGGCATGCTTTTCCTGCCG |  |
| 3MDH1G-U | aaacAGTATCCCCCAGCCGGACTG | Assembly of the gRNA cassette for *mdh1* editing |
| 3MDH1G-D | cacaCAGTCCGGCTGGGGGATACT |  |
| 1031mdh2g-U | aaacCAATGCCCCAGGCGGACTGG | Assembly of the gRNA cassette for *mdh2* editing |
| 1031mdh2g-D | cacaCCAGTCCGCCTGGGGCATTG |  |
| MDH2-GU1 | aaacGCGGTCTGGAGGACGACCTG | Assembly of the gRNA cassette for *mdh2* editing |
| MDH2-GD1 | cacaCAGGTCGTCCTCCAGACCGC |  |
| MDH2-GU2 | aaacGAACGAAGCCAGCGGTCTGG | Assembly of the gRNA cassette for *mdh2* editing |
| MDH2-GD2 | cacaCCAGACCGCTGGCTTCGTTC |  |
| MDH2-GU3 | aaacGCCATACAGGCCGACCTGGC | Assembly of the gRNA cassette for *mdh2* editing |
| MDH2-GD3 | cacaGCCAGGTCGGCCTGTATGGC |  |
| MDH2-GU4 | aaacTTGATGCCATGCCGCTGATC | Assembly of the gRNA cassette for *mdh2* editing |
| MDH2-GD4 | cacaGATCAGCGGCATGGCATCAA |  |
| MDH2-GU5 | aaacGTCGTGCGATAACCTGCGCA | Assembly of the gRNA cassette for *mdh2* editing |
| MDH2-GD5 | cacaTGCGCAGGTTATCGCACGAC |  |
| 1120IIAG-U | aaacCGTGGCCGCGCATCTGCGCA | Assembly of the gRNA cassette for *ptsF* editing |
| 1120IIAG-D | cacaTGCGCAGATGCGCGGCCACG |  |
| 1120PTSI-U | aaacGTATTTCCAGATTGGCCTGC | Assembly of the gRNA cassette for *ptsI* editing |
| 1120PTSI-D | cacaGCAGGCCAATCTGGAAATAC |  |
| g1-KIL-F | AAACCCATGGGGAGGCTCACGCGC | Assembly of the gRNA cassette for *lysR* editing in *K. intermedius* |
| g1-KIL-R | CACAGCGCGTGAGCCTCCCCATGG |  |

**Table S5.** gRNAs used in this study.

| **Gene** | **gRNA (N20, 5’-3’)** | **PAM** | **Relevance** |
| --- | --- | --- | --- |
| FKAID-U | GGCCCCTTCCAGTCCTGCGC | GGG | Inactivation of *frk* in *K. xylinus* and *K. rhaeticus* |
| FKAID-D | GCGCAGGACTGGAAGGGGCC |  |  |
| 1031mfs1g-U | GGCCGCCGAACCAAGCTGGC | GGG | Inactivation of *mtlT1* in *K. xylinus* |
| 1031mfs1g-D | GCCAGCTTGGTTCGGCGGCC |  |  |
| 1031mfs2g-U | CAAGCAGGAAGCCGACCTGC | TGG | Inactivation of *mtlT2* in *K. xylinus* |
| 1031mfs2g-D | GCAGGTCGGCTTCCTGCTTG |  |  |
| MFS3-grna-R | CGGCAGGAAAAGCATGCCAA | GGG | Inactivation of *mfs3* in *K. xylinus* |
| MFS3-grna-F | TTGGCATGCTTTTCCTGCCG |  |  |
| 3MDH1G-U | AGTATCCCCCAGCCGGACTG | GGG | Inactivation of *mdh1* in *K. xylinus* |
| 3MDH1G-D | CAGTCCGGCTGGGGGATACT |  |  |
| 1031mdh2g-U | CAATGCCCCAGGCGGACTGG | TGG | Inactivation of *mdh2* in *K. xylinus* |
| 1031mdh2g-D | CCAGTCCGCCTGGGGCATTG |  |  |
| MDH2-GU1 | GCGGTCTGGAGGACGACCTG | TGG | Base editing of *mdh2* in *K. xylinus* |
| MDH2-GD1 | CAGGTCGTCCTCCAGACCGC |  |  |
| MDH2-GU2 | GAACGAAGCCAGCGGTCTGG | AGG | Base editing of *mdh2* in *K. xylinus* |
| MDH2-GD2 | CCAGACCGCTGGCTTCGTTC |  |  |
| MDH2-GU3 | GCCATACAGGCCGACCTGGC | GGG | Base editing of *mdh2* in *K. xylinus* |
| MDH2-GD3 | GCCAGGTCGGCCTGTATGGC |  |  |
| MDH2-GU4 | TTGATGCCATGCCGCTGATC | GGG | Base editing of *mdh2* in *K. xylinus* |
| MDH2-GD4 | GATCAGCGGCATGGCATCAA |  |  |
| MDH2-GU5 | GTCGTGCGATAACCTGCGCA | AGG | Base editing of *mdh2* in *K. xylinus* |
| MDH2-GD5 | TGCGCAGGTTATCGCACGAC |  |  |
| 1120IIAG-U | CGTGGCCGCGCATCTGCGCA | CGG | Inactivation of *ptsF* in *K. xylinus* |
| 1120IIAG-D | TGCGCAGATGCGCGGCCACG |  |  |
| 1120PPPG-U | GTATTTCCAGATTGGCCTGC | CGG | Inactivation of *ptsI* in *K. xylinus* |
| 1120PPPG-D | GCAGGCCAATCTGGAAATAC |  |  |
| g1-KIL-F | CCATGGGGAGGCTCACGCGC | TGG | Base editing of *lysR* in *K. intermedius* |
| g1-KIL-R | GCGCGTGAGCCTCCCCATGG |  |  |

**Supplementary references**

(1) Huang, L. H.; Liu, Q. J.; Sun, X. W.; Li, X. J.; Liu, M.; Jia, S. R.; Xie, Y. Y.; Zhong, C. Tailoring bacterial cellulose structure through CRISPR interference-mediated downregulation of *galU* in *Komagataeibacter xylinus* CGMCC 2955, *Biotechnol. Bioeng.* **2020**, *117*, 2165–2176.

(2) Wang, Y.; Liu, Y.; Liu, J.; Guo, Y.; Fan, L.; Ni, X.; Zheng, X.; Wang, M.; Zheng, P.; Sun, J.; Ma, Y. MACBETH: multiplex automated *Corynebacterium glutamicum* base editing method, *Metab. Eng.* **2018**, *47*, 200–210.

(3) Wang, Y.; Zhao, D.; Sun, L.; Wang, J.; Fan, L.; Cheng, G.; Zhang, Z.; Ni, X.; Feng, J.; Wang, M.; Zheng, P.; Bi, C.; Zhang, X.; Sun, J. Engineering of the translesion DNA synthesis pathway enables controllable C-to-G and C-to-A base editing in *Corynebacterium glutamicum*, *ACS Synth. Biol.* **2022**, *11*, 3368–3378.
